# Genetic polymorphisms of *NAMPT* related with susceptibility to esophageal Squamous cell carcinoma

**DOI:** 10.1186/s12876-015-0282-6

**Published:** 2015-04-21

**Authors:** Chuanzhen Zhang, Daojie Yan, Shanshan Wang, Changqing Xu, Wenjun Du, Tao Ning, Changhong Liu, Meijuan Zhang, Ruiping Hou, Ziping Chen

**Affiliations:** 1 Digestive Department, Shandong Provincial Qianfoshan Hospital, Shandong University, Jingshi Road 16766#, Jinan, 250014 China; 2 Infectious Diseases Hospital, Laiwu Hospital, Taishan Medical College, Laiwu, China; 3 Key Laboratory of Carcinogenesis and Translational Research (Ministry of Education), Laboratory of Genetics, Peking University School of Oncology, Beijing Cancer Hospital & Institute, Beijing, China

**Keywords:** Nicotinamide phosphoribosyl transferase, Polymorphism, Haplotype, Esophageal squamous cell carcinoma, Susceptibility

## Abstract

**Background:**

Nicotinamide phosphoribosyl transferase (Nampt) plays a crucial role in tumorigenesis. The present study examines whether genetic polymorphisms of *NAMPT* are related to the risk of developing esophageal squamous cell carcinoma (ESCC).

**Methods:**

A total of 810 subjects were enrolled in this study, including 405 ESCC patients and 405 healthy controls. Using polymerase chain reaction-restriction fragment length polymorphism (PCR-RFLP), genotypes at rs61330082, rs2505568 and rs9034 of *NAMPT* were identified. Haplotypes were constructed using PHASE software. Multivariate logistic regression models were used to evaluate the potentiating effects of the genotypes, alleles and haplotypes on the development of ESCC.

**Results:**

The presence of genotypes CT and TT and allele T at rs61330082 was less frequent in ESCC cases than in controls (48.89% *vs.* 53.33%, *P* < 0.01, 95% CI: 0.33-0.68; 18.52% *vs.* 30.37%, *P* < 0.01, 95% CI: 0.22-0.50; 42.96% *vs.* 57.04%, *P* < 0.01, 95% CI: 0.38-0.61; respectively). No statistically significant differences existed in the distributions of genotypes or alleles at rs2505568 or rs9034 between ESCC cases and controls. Of five haplotypes constructed, haplotypes CTC, CTT and CAC were higher in ESCC cases (*P* < 0.01, OR = 1.57, 95% CI: 1.16-2.12; *P* = 0.04, OR = 1.72, 95% CI: 1.03-2.85; *P* < 0.01, OR = 3.39, 95% CI: 1.99-5.75; respectively) than in controls.

**Conclusion:**

Genetic polymorphisms of *NAMPT*, specifically genotype CC and allele C at rs61330082 as well as haplotypes CTC, CTT and CAC, were significantly correlated with ESCC susceptibility.

**Electronic supplementary material:**

The online version of this article (doi:10.1186/s12876-015-0282-6) contains supplementary material, which is available to authorized users.

## Background

Esophageal cancer is relatively common throughout the world. In 2008, approximately 482,300 new esophageal cancer cases were diagnosed and 406,800 deaths occurred [1]. Notably, almost 90% of cancer cases in the so-called “Esophageal Cancer Belt,” a region stretching from northern Iran through the Central Asian Republics to north-central China, are diagnosed as esophageal squamous cell carcinoma (ESCC). Common risk factors for ESCC include poor nutrition, a lack of adequate vitamin intake, tobacco smoking, excessive alcohol consumption, Barrett's Esophagus and mold pollution, among others [2]. In recent years, hereditary factors have also gained increasing attention for their role in the development of ESCC.

Nicotinamide phosphoribosyl transferase (Nampt) was first identified as pre-B-cell colony enhancing factor (PBEF). The *NAMPT* gene is located on chromosome7q22, spans 34.7 kb, has 11 exons and 10 introns, and produces cDNA of 2,357 kb translated into a 491-amino acid, 52-kDa protein that stimulates early B-cell formation [3]. Nampt was recently renamed “visfatin”, as it is a visceral, fat-derived adipokine that might mimic insulin function [4], and it may exist both intracellularly (iNampt) and extracellularly (eNampt). Nampt is also known to act as a rate-limiting enzyme in NAD biosynthesis, which is important because NAD availability is crucial for many vital cellular processes, including transcription regulation, DNA repair, cell cycle progression, apoptosis, calcium homeostasis, telomerase activity, antioxidation and oxidative stress, energy metabolism, circadian rhythm maintenance and chromatin dynamics regulation, and regulates factors of genomic stability and organismal metabolic homeostasis, including histone deacetylases (SirT1-T7), COOH-terminal binding proteins, CD38, poly(ADP-ribose) and polymerases [5,6]. Additionally, iNampt is involved in angiogenesis by activating the extracellular signal regulated kinase (ERK) 1/2 pathway and promoting the production of vascular endothelial growth factor (VEGF) and matrix metalloproteinase (MMP) 2/9 [7]. Independent of its enzymatic activity, eNampt plays a major role as a cytokine in the regulation of immune response [8]. Nampt is one of a few emerging adipokines (eg. leptin, adiponectin) whose expressions are correlated with the development of a variety of cancers [9]. Furthermore, a series of studies showed that Nampt might be a good biomarker of malignant potential and stage progression [8,10,11].

*NAMPT* shows a high degree of evolutionary conservation, suggesting that only tiny genetic changes can profoundly affect protein function and its dependent events. To date, the relationship between *NAMPT* genetic polymorphisms and disease has only been examined in bladder cancer, obesity and acute lung injury [12-14]. The aim of this study was to be the first to explore the relationship between *NAMPT* genetic polymorphisms and susceptibility to ESCC. Therefore, this case–control study was conducted using subjects recruited in Anyang, China, an area of high ESCC incidence. Three SNPs of *NAMPT*, including rs61330082 in the promoter region and rs2505568 and rs9034 in the 3’untranslated region (3’ UTR), were selected for this study because of their potential effects on the influence of Nampt expression.

## Methods

### Study subjects

A total of 405 ESCC patients were recruited from Anyang Tumor Hospital in Henan Province from February 2005 to July 2011. These subjects were diagnosed as having ESCC by qualified pathologists using endoscopic biopsies or surgical specimens, had no history of any other cancer and had not previously received chemotherapy or radiotherapy. The control group consisted of 405 gender- and age-matched (±1 year), healthy and genetically unrelated individuals recruited during the same time period from the same region. Each subject was required to sign an informed consent and complete a personal questionnaire, which included fields for demographic data and the related risk factors age, gender, tobacco smoking and alcohol consumption, prior to being included in this study. The ethic approval was provided by the ethic committee of Shandong Provincial Qianfoshan Hospital, Shandong University.

### DNA extraction

A 5-ml blood sample was collected from each subject, then genomic DNA was extracted using the Qiagen DNA Isolation Kit (Qiagen, Dusseldorf, Germany).

### Genotyping

Genotypes at rs61330082, rs2505568 and rs9034 of *NAMPT* were identified by polymerase chain reaction-restriction fragment length polymorphism (PCR-RFLP). Sequencing primers for rs61330082 and rs2505568 were used as previously described [12], and primers for rs9034 were designed using PRIMER 5.0 software (Canada). Information on primer sequences, the sizes of PCR products, restriction enzymes, enzyme digestion temperatures and restriction products is shown in Additional file 1: Table S1. PCR amplification was performed in a 20-μl reaction mixture containing 50–100 ng genomic DNA, 0.4 μl dNTPs (10 mM, Promega, USA), 0.8 μl each primer (10 mM, SinoGenoMax Co., Ltd.), 0.5 U Hotstart Taq DNA polymerase (5 U/μL, Qiagen, Dusseldorf, Germany) and 2 μl 10× PCR buffer. The PCR mixture was incubated for 2 min initial denaturation at 94.0°C followed by 36 cycles of 30 s denaturation at 94.0°C, 50 s annealing at the respective annealing temperatures (61°C, 52°C and 61°C, respectively) and 1 min extension at 72.0°C. The final extension was carried out for 10 min at 72.0°C. The PCR products were digested overnight by restriction enzymes at 37.0°C, then the digested products were analyzed following electrophoresis on a 3% agarose gel and photographing under UV light. To confirm the existence of polymorphisms, 10% of PCR products were directly sequenced. The representative pictures about the PCR-RFLP results and sequencing analysis were added in Additional file 2: Figure S1.

### Statistical analysis

Distribution of age between the cases and controls was compared using the Mann–Whitney *U* test, differences between the other demographic variables were compared using the McNemar test, and the observed genotype frequencies were tested for Hardy-Weinberg equilibrium using the chi-square test. PHASE 2.1 software (University of Chicago, USA) was used to construct haplotypes on the basis of the known genotypes and estimate haplotype frequencies. The possible effects of the genotypes, alleles and haplotypes on ESCC susceptibility were analyzed by odds ratio (OR) and 95% confidence interval (95% CI) using multivariate logistic regression models adjusted for age, gender, tobacco smoking and alcohol consumption. All tests were two-sided, and *P* values of < 0.05 were considered statistically significant. All statistical analyses were Zconducted using Stata 11.2 software (StataCorp., College Station, TX, USA).

## Results

### Demographic information of the subjects

The potential influence of age, gender, tobacco smoking and alcohol consumption on ESCC susceptibility was considered. As summarized in Table 1, the results showed that there were no significant differences in age or gender between groups or in the distributions of gender, tobacco smoking and alcohol consumption.

**Table 1:** **Demographic information of the subjects**

Characteristics	Cases (n = 405)		Controls (n = 405)		*P*
	n	%	n	%	
Age (year; mean ± SD)	60.89 ± 7.83		60.93 ± 7.91		0.87^a^
Gender					
Male	240	59.26	240	59.26	-
Female	165	40.74	165	40.74	
Tobacco smoking					
Ever	183	45.19	171	42.22	0.43^b^
Never	222	54.81	234	57.78	
Alcohol consumption					
Ever	117	28.89	96	23.70	0.11^b^
Never	288	71.11	309	76.30	

^a^Mann–Whitney *U* test.

^b^McNemar test.

### Correlation of *NAMPT* genotypes and alleles with ESCC susceptibility

All genotypes at the three *NAMPT* SNPs of the cases and controls were identified, and their distributions were found to be in Hardy-Weinberg equilibrium (*P* > 0.05). Table 2 shows that a significantly smaller proportion of the cases possessed either genotype CT or TT at rs61330082 than the controls (48.89% *vs.* 53.33%, *P* < 0.01; 18.52% *vs.* 30.37%, *P* < 0.01; respectively). Analogously, the frequency of allele T at rs61330082 in the cases was significantly decreased compared with the controls (42.96% *vs.* 57.04%, *P* < 0.01). Thus, subjects with genotypes CT or TT or allele Tat rs61330082 were less susceptible to ESCC (OR = 0.47, 95% CI: 0.33-0.68; OR = 0.33, 95% CI: 0.22-0.50; OR = 0.48, 95% CI: 0.38-0.61; respectively). Notably, no subjects carried genotype AA at rs2505568 or genotype TT at rs9034. With respect to the distributions at the two sites, no significant differences were found between the cases and controls (*P* > 0.05).

**Table 2:** **Correlation of** ***NAMPT*** **genotypes and alleles with ESCC susceptibility**

Genotype	Cases (n = 405)		Controls (n = 405)		*P* ^a^	OR^a^(95% CI)
	n	%	n	%		
rs61330082						
CC	132	32.59	66	16.30	1.00	1.00
CT	198	48.89	216	53.33	<0.01	0.47 (0.33-0.68)
TT	75	18.52	123	30.37	<0.01	0.33 (0.22-0.50)
C Allele	462	57.04	348	42.96	1.00	1.00
T Allele	348	42.96	462	57.04	<0.01	0.48 (0.38-0.61)
rs2505568						
TT	168	41.48	171	42.22	1.00	1.00
AT	237	58.82	234	57.78	0.91	1.02 (0.77-1.34)
AA	0	0.00	0	0.00	-	-
T Allele	573	70.74	576	71.11	1.00	1.00
A Allele	237	29.26	234	28.89	0.92	1.02 (0.77-1.34)
rs9034						
TT	0	0.00	0	0.00	-	-
CT	51	12.59	36	8.89	1.00	1.00
CC	354	87.41	369	91.11	0.09	0.64 (0.38-1.07)
T Allele	51	6.30	36	4.44	1.00	1.00
C Allele	759	93.70	774	95.56	0.13	0.70 (0.45-1.10)

^a^Conditional logistic regression adjusted for risk factors (tobacco smoking, alcohol consumption).

### Correlation of *NAMPT* haplotypes with ESCC susceptibility

Five haplotypes were constructed using PHASE software. As summarized in Table 3, the presence of haplotypes a (TTC) and b (TAC) was less frequent in the cases than in the controls (29.39% *vs.* 36.26%; 13.21% *vs.* 19.47%; respectively). Conversely, the presence of haplotypes CTC, CTT and CAC was more frequent in the cases than in the controls (36.17% *vs.* 30.91%; 4.81% *vs.* 2.94%; 14.92% *vs.* 8.92%; respectively). Each haplotype was then assessed for its ability to estimate susceptibility to ESCC (Table 4). Carriers with haplotypes TTC (−/a + a/a) or TAC (−/b + b/b) were less susceptible to ESCC (*P* < 0.01, OR = 0.61, 95% CI: 0.46-0.79; *P* < 0.01, OR = 0.69, 95% CI: 0.52-0.90; respectively) than those without these haplotypes (−/−). Conversely, individuals with haplotypes CTC, CTT or CAC (−/c + c/c, −/d + d/d or -e/e + e/e) were more susceptible to ESCC (*P* < 0.01, OR = 1.57, 95% CI:1.16-2.12; *P* = 0.04, OR = 1.72, 95% CI:1.03-2.85; *P* < 0.01, OR = 3.39, 95% CI:1.99-5.75; respectively) than those without these haplotypes (−/−).

**Table 3:** **Distributions of the estimated haplotype frequencies**

Haplotypes	SNP positions			Cases (n = 405)		Controls (n = 405)	
	rs6133082	rs2505568	rs9034	n	%^a^	n	%^a^
a	T	T	C	238	29.39	294	36.26
b	T	A	C	107	13.21	158	19.47
c	C	T	C	293	36.17	250	30.91
d	C	T	T	39	4.81	24	2.94
e	C	A	C	121	14.92	72	8.92

^a^Conditional calculated by PHASE software.

**Table 4:** **Correlation of** ***NAMPT*** **haplotypes with ESCC susceptibility**

Haplotype	Cases (n = 405)		Controls (n = 405)		*P* ^a^	OR^a^(95% CI)
	n	%	n	%		
a = TTC						
−/−^b^	258	63.70	204	50.37	1.00	1.00
-/a + a/a	147	36.30	201	49.63	<0.01	0.61 (0.46-0.79)
b = TAC						
−/−	237	58.52	195	48.15	1.00	1.00
-/b + b/b	168	41.48	210	51.85	<0.01	0.69 (0.52-0.90)
c = CTC						
−/−	111	27.41	147	36.30	1.00	1.00
-/c + c/c	294	72.59	258	63.70	<0.01	1.57 (1.16-2.12)
d = CTT						
−/−	354	87.41	372	91.85	1.00	1.00
-/d + d/d	51	12.59	33	8.15	0.04	1.72 (1.03-2.85)
e = CAC						
−/−	336	82.96	381	94.07	1.00	1.00
-/e + e/e	69	17.04	24	5.93	<0.01	3.39 (1.99-5.75)

^a^Logistic regression model, adjusted for age, gender, tobacco smoking and alcohol consumption.

^b^The minus sign (−) denotes any haplotype. For example: −/a indicates the a haplotype in combination with any other haplotype.

## Discussion

Esophageal cancer has a poor prognosis, and in 2009, its mortality ranked the fourth and its incidence fifth among all reported cancers in China [2]. While esophageal cancer has been studied in depth, the specific mechanism by which it develops is still unclear. Given the known influence of genetic polymorphisms on certain types of cancer, it was important for this group to analyze the as yet unknown association between genetic polymorphisms and ESCC susceptibility, particularly since we are located within a high-ESCC-incidence region of China. For this study, *NAMPT* was selected as a basis for analyzing this association.

The adipokine Nampt was first reported as a pleiotropic protein, and is widely known as a key regulator of NAD, which is intimately involved in proliferation, cytokine production, immunological regulation and angiogenesis. Nampt is overexpressed in a variety of cancers, including that of the stomach and colorectal cavity [15,16], and its inhibitor FK866 is a widely studied anti-cancer agent [17].

In this study, the results demonstrated that the presence of genotypes CT and TT and allele T at rs61330082 of *NAMPT* was significantly decreased in the cases compared with the controls. It was therefore suggested that these genotypes or this allele might reduce ESCC susceptibility. In other words, genotype CC or allele C at rs61330082 might increase carrier susceptibility to ESCC. Considering the rs61330082 loci in the promoter region, its genetic mutation might influence the structure or function of Nampt protein. This finding is therefore commensurate with the probable roles of *NAMPT* in tumorigenesis and its elevated expression in gastric and colorectal cancers [15,16]. Although its expression did not vary in ESCC [18] and its genetic polymorphisms were never studied in ESCC, this result was consistent with a similar bladder cancer study by Zhang et al. [12].

Genotype AA at rs2505568 and genotype TT at rs9034 could not be detected in this study, which was a similar finding to Zhang’s previous study [12]. Whether the limited sample size in that study was responsible for the absence of these genotypes in bladder cancer deserves further research. In spite of the importance of the 3’ UTR on the regulation of gene expression, polymorphisms at rs2505568 and rs9034 were found to be independent risk factors for the development of ESCC, also partially coinciding with Zhang’s study [12], although pathogenesis between ESCC and bladder cancer is comparatively limited.

Haplotype analysis assessed disease susceptibility more powerfully by analyzing the combined action of multiple loci. From the selected genetic sites, five haplotypes were constructed. The presence of haplotypes CTC, CTT or CAC was positively correlated with the development of ESCC, while the presence of haplotypes TTC or TAC protected carriers from ESCC. The determinant impact of allele C at rs61330082 among the three SNPs is thus implied.

This study had certain limitations worth noting. Specifically, the findings need to be confirmed by a larger sample of the population from more high-ESCC-incidence regions, and there are many more convenient and effective methods of confirming genotypes than PCR-PFLP. Additionally, functional studies and gene-environment interaction studies are needed to offer more authentic and integrative proof about the influence of *NAMPT* on ESCC.

## Conclusions

In summary, genetic polymorphisms of *NAMPT* were significantly correlated with ESCC susceptibility in the studied Chinese population. Genotype CC and allele C at rs61330082 as well as haplotypes CTC, CTT and CAC were each risk factors for ESCC. Thus, these findings might provide one or more novel diagnostic indicators for ESCC.

## Additional files

Additional file 1: Table S1. Primer sets used for the three genetic polymorphisms. Additional file 2: Figure S1. The representative pictures about the electrophoresis patterns of PCR-RFLP results and sequencing analysis.

## Abbreviations

ESCC: Esophageal squamous cell carcinoma
Nampt: Nicotinamide phosphoribosyl transferase
PBEF: Pre-B-cell colony enhancing factor
ERK: Extracellular signal regulated kinase
VEGF: Vascular endothelial growth factor
MMP: Matrix metalloproteinase
PCR-RFLP: Polymerase chain reaction-restriction fragment length polymorphism
