# Supplementary material for: Genetic polymorphisms of NAMPT related with susceptibility to esophageal Squamous cell carcinoma
Source: BMC Gastroenterol. 2015 Apr 21;15:49. doi: 10.1186/s12876-015-0282-6 (PMC4408598; doi:10.1186/s12876-015-0282-6)
Supplement: Additional file 1: Table S1. — Primer sets used for the three genetic polymorphisms. [file 12876_2015_282_MOESM1_ESM.docx]

Table S1 Primer sets used for the three polymorphisms

| SNP Posion | Primers(forward and reverse) | PCR Products | Restriction Enzymes | Ristriction Products |  |
| --- | --- | --- | --- | --- | --- |
| rs61330082 | F:5’-GAGGCATGGCTGAGACTTCTA-3’ | 201bp | ScrFI (37℃) | TT:201bp |  |
|  | R:5’-TGTTTCAAACCTCGTTGCTG-3’ |  |  | CC:135bp,66bp |  |
|  |  |  |  | CT:201bp,135bp,66bp |  |
| rs2505568 | F:5’-AAGCTTTTAGGGCCCTTTG-3’ | 230bp | RsaI (37℃) | AA:230bp |  |
|  | R:5’-TCATGAAAAGTTGGAAAGACTGTT-3’ | | | TT:163bp,67bp |  |
|  |  |  |  | AT:230bp,163bp,67bp |  |
| rs9034 | F:5’-CCCACTACTCCAGCCTAAGG-3’ | 186bp | MluCI(37℃) | CC:186bp |  |
|  | R:5’-TAGCCTCCTCCCTTCCCTAG-3’ |  |  | TT:95bp,91bp |  |
|  |  |  |  | CT:186bp,95bp,91bp |  |
| *F and R indicate forward and reverse primers, respectively. | | | | |  |
