# Supplementary material for: Genetic polymorphisms of NAMPT related with susceptibility to esophageal Squamous cell carcinoma
Source: BMC Gastroenterol. 2015 Apr 21;15:49. doi: 10.1186/s12876-015-0282-6 (PMC4408598; doi:10.1186/s12876-015-0282-6)
Supplement: Additional file 2: Figure S1. — The representative pictures about the electrophoresis patterns of PCR-RFLP results and sequencing analysis. [file 12876_2015_282_MOESM2_ESM.docx]

**Electrophoresis patterns of PCR–RFLP results**

**rs61330082**

**CC CC TT CC CT CT**


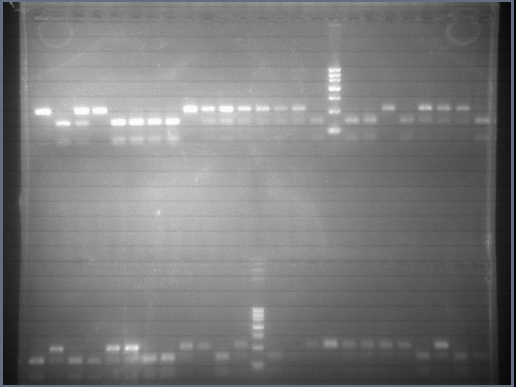


**rs2505568**

**AT TT AT TT AT AT**

**
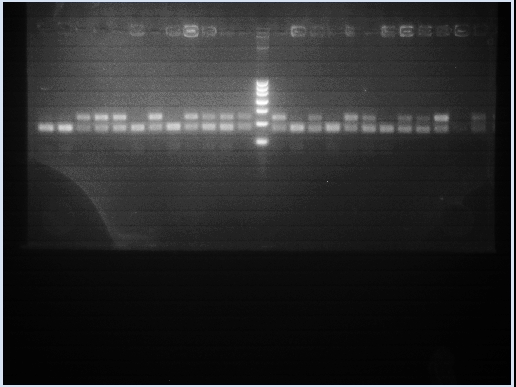
**

**rs9034**

**CC CC CT CC CC CC**

**
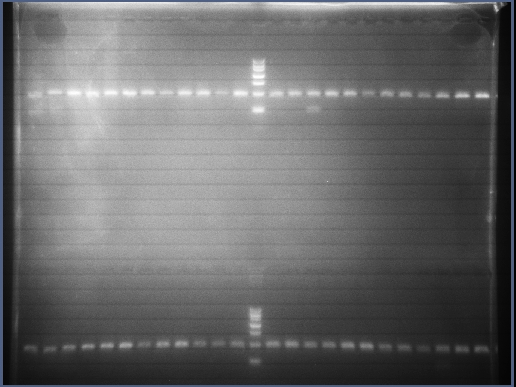
**

**Sequencing maps of PCR products**

**
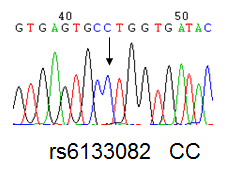
**

**
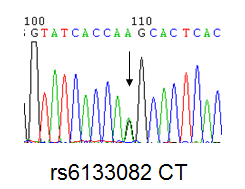
**

**
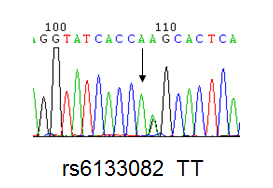
**

**
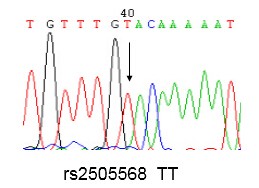
**

**
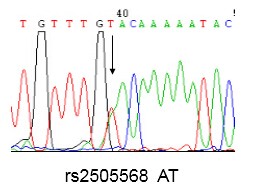
**

**
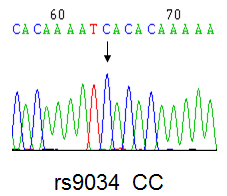
**

**
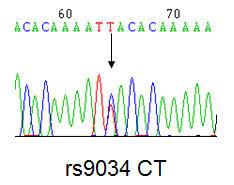
**
